# Primary care and abortion provider perspectives on mail-order medication abortion: a qualitative study

**DOI:** 10.1186/s12905-024-03202-z

**Published:** 2024-07-03

**Authors:** Sarah Raifman, Tanvi Gurazada, Jessica Beaman, M. Antonia Biggs, Eleanor Bimla Schwarz, Marji Gold, Daniel Grossman

**Affiliations:** 1 Advancing New Standards in Reproductive Health, University of California, San Francisco, 1330 Broadway Suite 1100, Oakland, CA 94612 USA; 2 Nell Hodgson Woodruff School of Nursing at Emory University, 1520 Clifton Rd, Atlanta, GA 30322 USA; 3 San Francisco Division of General Internal Medicine, University of California, 1001 Potrero Ave, San Francisco, CA USA; 4 Montefiore Medical Center, Albert Einstein College of Medicine, 3544 Jerome Ave, Bronx, NY 10467 USA

**Keywords:** Medication abortion, Mail-order pharmacy, Qualitative, Primary care

## Abstract

**Background:**

This qualitative study aims to assess perspectives of clinicians and clinic staff on mail-order pharmacy dispensing for medication abortion.

**Methods:**

Participants included clinicians and staff involved in implementing a mail-order dispensing model for medication abortion at eleven clinics in seven states as part of a prospective cohort study, which began in January 2020 (before the FDA removed the in-person dispensing requirement for mifepristone). From June 2021 to July 2022, we invited participants at the participating clinics, including six primary care and five abortion clinics, to complete a semi-structured video interview about their experiences. We then conducted qualitative thematic analysis of interview data, summarizing themes related to perceived benefits and concerns about the mail-order model, perceived patient interest, and potential barriers to larger-scale implementation.

**Results:**

We conducted 24 interviews in total with clinicians (13 physicians and one nurse practitioner) and clinic staff (*n* = 10). Participants highlighted perceived benefits of the mail-order model, including its potential to expand abortion services into primary care, increase patient autonomy and privacy, and to normalize abortion services. They also highlighted key logistical, clinical, and feasibility concerns about the mail-order model, and specific challenges related to integrating abortion into primary care.

**Conclusion:**

Clinicians and clinic staff working in primary care and abortion clinics were optimistic that mail-order dispensing of medication abortion can improve the ability of some providers to provide abortion and enable more patients to access services. The feasibility of mail-order pharmacy dispensing of medication abortion following the Supreme Court *Dobbs* decision is to be determined.

**Trial registration:**

Registry: Clinicaltrials.gov. Trial registration number: NCT03913104. Date of registration: first submitted on April 3, 2019 and first posted on April 12, 2019.

**Supplementary Information:**

The online version contains supplementary material available at 10.1186/s12905-024-03202-z.

## Background

Medication abortion, using mifepristone and misoprostol, is now the most common type of abortion in the United States (US) [1]. The US Food and Drug Administration (FDA) approved mifepristone for termination of pregnancy in 2000 with the caveat that it could only be dispensed in-person in a facility setting by an authorized provider, which was later codified in the drug’s Risk Evaluation and Mitigation Strategy (REMS). The requirement that mifepristone be dispensed in person was in place from 2000 until 2021. In 2021, the FDA undertook a full review of the mifepristone REMS program and determined that in-person dispensing was not necessary to assure the safe use of mifepristone for abortion through 70 days’ gestation [2]. Misoprostol, the second of the two medications used together to induce abortion, is not restricted.

Medication abortion is a safe and effective treatment that can be provided in various clinical settings, including via telemedicine, according to current guidelines (as of May 2024) [3, 4]. Key clinical considerations and steps for prescribing medications for abortion include reviewing a patient’s history, determining gestational duration eligibility, assessing for contraindications, and providing education and counseling about the process, including warning signs. Follow-up assessment to determine if the abortion is complete can be virtual or in person. Since the FDA decision in 2021, patients have been able to access medication abortion entirely via telemedicine which has been found to be safe, effective, and comparable to in-person care [4, 5]. Despite this evidence, a case before the Supreme Court in 2024 challenges the FDA’s 2016 and 2021 actions with respect to mifepristone’s approved conditions of use, including the decision to remove the in-person dispensing requirement.

The previous REMS’ in-person dispensing requirement for mifepristone meant that clinics had to stock and monitor medications at their facilities, which was an impediment for providers, particularly primary care clinicians and administrators, to offering medication abortions services. For the last 20 years, medication abortion has been offered primarily at clinics that provided relatively large numbers of abortions, and where abortion care was a focus of the practice. Primary care providers are the first point of care for many patients and are uniquely positioned to deliver office-based reproductive health services, including abortion. Evidence shows that general internists, family medicine physicians, nurse practitioners, physician assistants, and certified nurse midwives can safely provide medication abortion services [6]. However, few actually do so in primary care settings; a national study of family physicians found that three years after completing residency only 3% reported providing abortion [7]. Primary care physicians have reported that the REMS required involvement of clinic administrators, who may not be supportive, and that its complexity led many to believe that offering medication abortion was not worth the effort [8]. Additionally, restrictions on federal funding complicates the ability to provide abortion care for primary care providers working in federally qualified health centers [9].

The potential benefits of mail-order pharmacy dispensing of mifepristone are numerous [10]. Mail-order pharmacy dispensing could increase the number and type of clinicians willing and able to provide medication abortion services by reducing the challenges and costs of stocking the medications in their clinics. No other medications prescribed in primary care are required to be dispensed in person. There has been substantial growth in the use of mail-order pharmacies to fill routine outpatient prescriptions in the US [11]. Medications are routinely prescribed to the patient’s preferred pharmacy, which may be either a local retail pharmacy or a mail-order pharmacy, and many medications are cheaper for patients when obtained via mail-order pharmacy. Mail-order dispensing of mifepristone and misoprostol could enable access to abortion care earlier in pregnancy, by helping patients bypass obstacles to clinic-based care, and it could reduce abortion stigma. Research shows that patients are interested in accessing abortion pills in alternative ways, including buying abortion pills online and having them sent by mail [12]. Preliminary studies indicate that mail-order dispensing of mifepristone is safe, effective, and acceptable to patients [13] and that screening for medication abortion care eligibility by history alone without ultrasonography or pelvic examination maintains high effectiveness and low risk [14].

In January 2020, we launched a prospective cohort study of mail-order pharmacy dispensing of medications for abortion, before the FDA removed the in-person dispensing requirement for mifepristone (temporarily in April 2021 and then permanently in December 2021) [13]. Under an Investigational New Drug application to the FDA, we aimed to evaluate the feasibility, acceptability, and effectiveness of dispensing mifepristone and misoprostol by mail after an in-person clinical assessment. We enrolled 538 patients from January 2020 through May 2022 (excluding a pause in recruitment from February to May 2020 due to the COVID-19 pandemic). Interim quantitative results have been published previously [15]. To inform future implementation and scale-up of mail-order pharmacy dispensing of mifepristone, we conducted interviews with clinicians and staff involved in the prospective cohort study from June 2021 to July 2022 to better understand their experiences.

## Methods

Details of the prospective cohort study were recently published [15, 16]. Briefly, we collaborated with 11 clinics that were interested in offering a mail-order pharmacy dispensing model for medication abortion. Clinics included six primary care sites, four of which had not provided abortion care prior to the study, and five abortion clinics. Participating clinics were located in states with political contexts surrounding abortion that ranged from very supportive to hostile, including California, Colorado, Delaware, Georgia, New York, Pennsylvania, and Rhode Island. The study was advertised at meetings and on listservs that included primary care and abortion clinicians and clinic-based staff across the country. Interested sites were informed that the study would provide training in medication abortion (if needed) and support integrating medication abortion services into practice. Sites with sufficiently large eligible patient populations and administrative support were selected to participate. Despite the 2016 mifepristone label update, which removed a prior requirement of direct observation of mifepristone ingestion in clinic [15], all participating abortion clinics continued to require that patients swallow the mifepristone in clinic when this study was launched in 2020. One abortion site had participated in another research study that offered medication abortion via telemedicine. All clinicians and clinic-based staff (referred to together as “providers”) at participating sites were invited to a training led by the research team prior to launching patient recruitment, which included information on medication abortion provision (for new providers) and on study procedures, including informed consent, enrollment, prescribing, and follow-up.

Clinical care provided in this study matched current clinical guidelines [3, 4]. Clinicians assessed patients’ eligibility in person for medication abortion and this study. Clinicians determined patients’ gestational duration using ultrasound or clinical assessment (which included reported date of last menstrual period, other clinical history, and physical exam), depending on the clinic. Clinic staff consented and enrolled patients, and clinicians sent prescription orders to an existing mail-order pharmacy that agreed to participate in the study. The pharmacy filled prescriptions and sent discrete packages to patients by mail as soon as possible (usually the same day or next business day). Patients received the medications at their preferred address (options included their residence, office, friend or family member’s residence, or the recruitment clinic) and took the pills as instructed by the provider. Clinicians followed up with participating patients in person or by telephone.

We invited all clinicians and clinic-based staff at all sites who completed a training with our team and were involved in evaluating, enrolling, or prescribing medication for patients in the study to participate in semi-structured video interviews about their experiences at the end of recruitment. We purposively sampled participants until thematic saturation was reached with respect to perceived benefits and barriers of mail-order pharmacy dispensing (i.e., until no new benefits or barriers were discussed in interviews). We aimed to conduct 2–3 interviews per site to capture a variety of perspectives within each site. The interviewer (TG), a non-clinician member of our research team trained in public health, qualitative interviewing and research methods and not involved in the main study, reached out directly to potential participants by email and invited them to schedule an interview; if the interviewer did not receive a response within one week, she followed up two more times by email (three times total).

We conducted one-on-one video interviews over Zoom using a semi-structured interview guide between June 2021 and July 2022. Interviews explored perceived benefits and concerns with mail-order pharmacy dispensing, patient interest in the model, and barriers to potential implementation beyond the study context. We developed the interview guide specifically for this study and used two slightly different versions, one for clinics who were new to providing abortion (Supplement 1) and one for clinics who were experienced with abortion care (Supplement 2). Interview participants verbally confirmed their informed consent to participate immediately prior to the interview. The interviewer asked permission to audio-record the interviews, which were later professionally transcribed for analysis. We did not compensate clinicians and clinic staff for participating in an interview. The study was approved by the University of California, San Francisco Institutional Review Board.

To analyze the data, we used a version of thematic analysis proposed by Nowell et al. [17], which consists of becoming familiar with the data, generating initial codes, searching for themes, reviewing themes, defining and naming themes, and producing a summary report. The interviewer (TG) and first author (SR) read all transcripts and then they each re-reviewed a separate set of transcripts to develop preliminary codes, consisting of both a priori codes drawn from the semi-structured interview guide and relevant subcodes that emerged from the data. The two coders met to discuss their approach, to define and refine codes, and to reconcile any discrepancies in codes. After finalizing the code book and method of applying the codes to the text, at least one author coded each of the transcripts in Dedoose. We then identified themes and sub-themes, summarized them, and compared data across site types (abortion and primary care clinics). Throughout the manuscript writing process, we further honed descriptions and names of themes. In presenting results, we accompany quotes with a participant identification (ID) number (in order of appearance) and site type (abortion versus primary care clinic).

## Results

Between June 2021 and July 2022, we invited 31 clinicians and staff to participate in interviews and conducted a total of 24 interviews with clinicians (13 physicians and one nurse practitioner) and clinic staff (10 administrators and counselors). Two invitees declined to participate, one was on parental leave, and four others did not respond to our invitations. We interviewed 1–5 people from each clinical site. Of the 24 participants, 12 worked at primary care sites new to abortion provision, and 12 worked at abortion clinics. Interviews averaged 32 min in length (range 19–45 min). In response to interview questions about implementing the mail-order model, respondents identified key perceived benefits (including the normalizing of abortion, expansion of abortion services in primary care, increased patient autonomy and privacy, improved access to care, lack of need to stock medications in clinic, and improved efficiency of clinic flow) as well as key concerns (pertaining to logistics, clinical care, feasibility, and institutional barriers).

### Normalizing abortion

Primary care respondents highlighted that mail-order dispensing would contribute to normalizing and destigmatizing abortion care. Being able to offer abortion services felt aligned with the scope of practice of primary care providers. After being asked about what it was like to offer mail-order medication abortion services, one primary care provider explained:

*“…the biggest thing about it is it just feels very normal. Like I said, it just feels very normal for me to sit at the computer and be like, okay, I sent everything to the pharmacy. Which is what I would do with anything else, as opposed to be having to sit there and hand someone a pill and watch them take it.”* (ID4, primary care clinic).

Another primary care provider said, “*this really felt like it’s the wheelhouse of internal medicine. Really, if we stop calling it ‘training to provide medication abortion’, that sounds really hard, ‘prescribe these pills’ is like the language shift. Of course, why would I not prescribe these pills?*” (ID1, primary care clinic**)**. Another described how it makes the prescribing process more similar to other types of care:

“*I think sending something to a pharmacy feels … within how medicine works for other things, and more similar to any other prescription that I send in where there might be like a blood test I want to check or an ultrasound I want to get, and then as soon as I have that, I’ll send in something.”* (ID6, primary care clinic).

Experienced abortion providers also acknowledged that mail-order pharmacy dispensing could help to remove unnecessary stigma and anxiety for patients. One provider explained:

“…*it adds to destigmatizing as well. … it shows patients that ‘oh this can just be mailed to my house. This isn’t some gate-kept high-security medication that is dangerous and going to kill me’ or whatever. This is only being kept behind bars because of the stigma and because of the way that our country is run. There’s no actual reason for these medications to be as difficult to obtain as they are*.” (ID12, abortion clinic).

### Expansion of abortion services in primary care

Primary care site respondents noted that offering abortion services at their facilities allowed them to provide continuity of care for their patients without having to refer them to outside providers. Prior to participating in the study, it was challenging for some clinicians and staff to explain to their patients why they were not able to offer abortion services after having counseled them about their pregnancy options (including abortion). One person explained:

“*… patients would ask us like, ‘Oh, great. You counseled us about my options. I want a medication abortion. Wait a second, if you’re a doctor and it’s a pill, why can’t you prescribe that?’…. you know, it just felt really crappy not to be able to do that. Like I said, many of our patients have a lot of barriers anyhow for their healthcare; a lot of them language-based and financial, because we’re in a Hyde state [a state without public insurance coverage for abortion], so people are having to pay for their abortions in addition. So, it just seemed like a needless additional barrier to put our patients through.”* (ID1, primary care clinic).

Respondents said that being able to offer abortion services was fitting for primary care, which involves treating a range of patient health concerns. One provider recalled:

“*I had one memorable patient who would come in for knee pain …and just incidentally had a positive pregnancy test…so then we shifted the visit from knee injury to taking care of the unintended and undesired pregnancy. We had another patient who came in for a [contraceptive] implant placement who was pregnant so then we ended up pivoting.*” (ID2, primary care clinic).

Another respondent expressed concern about patients not receiving the care they desired when she referred them to a local abortion clinic and had difficulty following up with them:

“*I would just keep thinking about how we’re doing 3 to 4 of these per month and where have all these people been going for their care prior to this time? Are they just disappearing, are they going to Planned Parenthood? Are they not getting care? Who knows*?” (ID4, primary care clinic).

Primary care providers underscored that the relationships they build with patients over time position them to be able to provide quality abortion care for their patients. One said:

“*I really love the idea of people being able to get abortion care from within a practice in which they already know the people, they’re already familiar with, they feel very safe, and it feels like part of the regular care because it is.*” (ID5, primary care clinic).

Another explained how abortion services fit within the other reproductive health services they offer:

*“I think it just feels like part of primary care in all honesty, that you’re seeing patients and asking about contraception, family planning, pregnancy desires, wanting to not be pregnant… [that you can] provide that service especially for patients on my panel who might be immigrants, or refugees, or experiencing substance use or other barriers…”* (ID6, primary care clinic).

Some respondents mentioned concerns about the potential for some patients—particularly those already facing barriers to healthcare access—to get lost during the referral process. A primary care participant said:

“*It didn’t feel good to always have to refer a patient out to get an abortion if that’s what they chose, especially when we have patients with lots of social challenges that have lots of barriers to access and have poor health literacy. It did mean that certain patients every now and then would fall through the cracks, or maybe not be able to get services fast enough to be able to do medication abortion and then have to do surgical abortion.*” (ID3, primary care clinic).

### Increased patient autonomy and privacy

Clinicians and clinic staff at both primary care and abortion clinics recognized that the mail-order option afforded their patients more control and autonomy in how they received and took the mifepristone. As of 2016, the drug’s label no longer required that patients swallow the mifepristone medication in clinic, yet participants at abortion clinics described how their facility still required this practice due to existing clinic precedent and the mail-order option allowed them to circumvent it. According to some clinicians and clinic staff, providing patients with the option of where and when to take the mifepristone was about showing them respect, which helped to put patients at ease and make them feel empowered. One respondent, whose clinic still required that patients take the mifepristone at the facility (despite the 2016 drug label update which removed this requirement), said:

“*I wish we had the option that we could mail our patients medication all the time because …they were so stoked. You could tell it totally changed people’s experience. …it comes down to that sense of control, just being trusted with the administration of your own medication. It’s very infantilizing to have to be like, ‘No, the doctor has to put this pill in your hand and watch you take it’, when there’s no medical reason for it to be done that way. I could definitely see the benefits of this being pretty wide-reaching.*” (ID7, abortion clinic).

Others agreed, saying that increased autonomy, resulting from being able to choose when and where to start the abortion process, could allow patients to have enhanced physical comfort and emotional support during the abortion.

Finally, participants from sites that were still requiring patients to swallow the mifepristone in person explained that being able to choose when and where to take the pills and pass the pregnancy is essential to some patients’ ability to have an abortion at all. One provider explained:

*“…to have that flexibility on the other end was really, really nice for a lot of our patients who like absolutely need those full five or six days of work to make their rent at the end of the month.*” (ID5, primary care clinic).

Several providers articulated that mail-order dispensing is valuable because it gives patients more options. One person explained, “*I think the biggest benefit is that it offers another option for patients. The more options you can offer patients, the more they can pick the one that most suits their life and their needs at that moment*” (ID8, primary care clinic).

Participants also noted that mail-order dispensing, presumably if implemented in combination with telemedicine (though telemedicine was not a part of this study), has the potential to help reduce stress, maintain privacy about the pregnancy decision, and avoid exposure to protesters outside of the clinic. One provider said:

*For patients, just the sheer fact of coming to a clinic can have stress connected to it, whether it’s exposure to protestors, whether it’s just the nature of being in a medical place. That can cause anxiety in any of us. So, not having to do that is potentially a stress reduction…not having to tell anyone or see anyone …that’s a big part of it as well.”* (ID9, abortion clinic).

Another respondent compared mail-order pharmacy dispensing to brick-and-mortar pharmacist dispensing, commenting on the increased privacy that comes with the former: *“…not having to go to the pharmacy with a bunch of scripts … and have the pharmacist be like ‘hmm, what are these for?’ That’s also really nice to add that level of privacy*” (ID10, abortion clinic). While generally providers believed that mail-order dispensing would improve privacy for patients who choose it, one provider did recognize that some patients may not prefer mailing mifepristone to a personal address for privacy reasons, “*It didn’t come up too often, but we did have a few people who weren’t able to send the medications for privacy reasons to the place that they were living*” (ID11, abortion clinic).

### Improved access to care

Though none of the study clinics implemented a fully remote care model for this study, participants from both abortion and primary care clinics spoke to how the mail-order model had the potential to improve access to care if employed together with telemedicine. Many providers highlighted the potential for a fully remote model, which would include telemedicine evaluation for eligibility followed by mail-order pharmacy dispensing of medications. In response to being asked about advantages of the model, one respondent said:

*Definitely access. I mean, just the realities of getting to clinic are really significant for a lot of people. So, getting there, the time that it takes to get there, the time that it takes to get work covered, childcare, all of these different things. …even if it’s actually not that hard, there’s no reason for it. It doesn’t even have to be insurmountable; it’s just like, if you don’t need to do it, you don’t need to do it.* (ID9, abortion clinic)

One participant from an abortion clinic said:

“*I would love to see it go a step further where they wouldn’t have to come to the clinic at all. … I see lots of good potential there. I love [mail-order]. I’d love to just do a telehealth model with it. It would increase access exponentially.*” (ID13, abortion clinic).

A provider at a primary care facility said:

“*the big hope is that they wouldn’t have to come in person at all … I remember that was our first question. I was like, ‘Oh my gosh, can we do this all over telemedicine because wouldn’t that be amazing?’ …I think mail-order … really supports the idea of telemedicine.*” (ID8, primary care clinic).

Another primary care provider said, *“…taking the next step and making this part of a telemedicine visit would be huge. And that’s where I think the model would be really a big benefit in opening up access to patients*” (ID14, primary care clinic). Mail-order pharmacy dispensing seemed to be inextricably linked to telemedicine for one provider who referred to “*true mail-order*” as a model where “*a patient never even needs to come to the clinic*” (ID9, abortion clinic).

Participants more experienced in providing abortion care added that combining mail-order pharmacy dispensing with telemedicine could enable them to see more patients each day and to hire providers in other locations, which could expand the workforce. One respondent said:

“*If we were able to offer that up front without them actually having to come into the clinic to begin with, that’s the goal. That’s life-changing. That changes the way that we run the clinic. That changes our capacity for a day and how many abortions we’re able to provide, versus how many people are actually in the building. … I’m sure we could add telemedicine onto a schedule that’s already packed for in-person appointments. It frees up space for us and for the patient.*” (ID12, abortion clinic).

Another respondent explained:

“*Most of our doctors have day jobs and work with us when they can. [Mail-order pharmacy dispensing and telemedicine] would increase that availability and the flexibility of scheduling with more people able to work from where they are…I do see it as increasing access and just ease of providing care overall.*” (ID13, abortion clinic).

### Lack of need to stock medications in clinic

Primary care respondents in particular highlighted that the mail-order pharmacy dispensing model was beneficial because it facilitated offering abortion care without requiring them to take on the logistical, financial, and regulatory responsibilities of purchasing, stocking, and tracking mifepristone in their clinics. One primary care provider said:

“*We didn’t have to worry about: are we stocked with mifepristone, what is the expiration date, when’s the next shipment arriving… knowing that I could send a prescription as easily as I do a cholesterol [medication] prescription was a really nice backup to have.*” (ID5, primary care clinic).

Another provider explained that avoiding stocking medications in clinic created a more streamlined administrative approval process within their own facility for initiating abortion provision:

*“I think I would have had an additional political barrier to be able to get permission to order and stock in my clinic. I think that would have been a no-go because that would have been the ‘you have to run it by the CEO thing’ and having it be mail-order just took that outside of the clinic. That is the number one best thing.”* (ID1, primary care clinic).

Even for providers at abortion clinics, where systems for stocking and tracking mifepristone were already in place, the mail-order model simplified the process. One participant explained, “*Administratively, there was a small benefit. We didn’t have to order pills, we didn’t have to wait for pills to come in, we didn’t have to log them in, those kinds of things*” (ID15, abortion clinic). Another abortion provider explained that relying on a pharmacy to dispense the medications might also lead to improved quality control:

“*I think it could definitely streamline the process. … that would be great having to not keep track of [mifepristone], how much we have, and how we use it. At the end of some days, it just feels like we’re counting everything a thousand different times, and so having to remove that from the rotation would be really nice and not having to worry that it would ever be stolen for any reason. Then, like I said with the batch, storing it in a place where it doesn’t get too hot or too cold, you don’t have to worry that way.”* (ID10, abortion clinic).

### Improved efficiency

Respondents also commented on how the mail-order pharmacy dispensing model could increase the ease and efficiency of daily clinic flow. One participant from an abortion clinic explained:

“*For the health center, I think it took a lot of burden off of having to dispense that medication and keep that patient longer in the clinic to talk through each of those medications, have them take that medication. Depending on how busy the clinics are … definitely opens up a lot of avenues to save time and also save hands of who’s available to do what.*” (ID11, abortion clinic).

At one site, where medication abortion was available prior to the study but the care team had to go to the facility’s pharmacy to obtain the medications for the patient, providers said the mail-order option was preferable for them because it saved them time. They said:

“*In a sense…it takes less time and hassle because, having someone have to go down to the pharmacy to grab the medication, and watch them take it and record the time …it seems a little excessive for basic healthcare medication. I wish we didn’t have such restrictions.*” (ID16, primary care clinic).

A couple experienced providers noted that the model could reduce the amount of time a patient needs to spend physically in the clinic. One said, “*Sometimes, depending on how busy we are, they could be waiting for a few hours. So, with this process, it might be able to cut down the time they’re just sitting and waiting*” (ID10, abortion clinic).

### Logistical concerns

In addition to identifying perceived benefits, interview participants discussed several potential challenges about mail-order pharmacy dispensing of mifepristone, including potential logistical concerns with sending prescriptions to the pharmacy and delivering packages to patients in a timely and confidential manner. Potential shipping delays were top of mind for most providers, though few experienced real challenges around delays. Providers were concerned that delays could impact when patients were able to initiate medication abortion. One provider said, “*we had two or three patients that, because of the weather and because of the day we prescribed, weren’t able to do the medication abortion when they wanted to*” (ID18, primary care clinic).

Providers described a learning curve with regard to expediting the prescribing process and setting accurate expectations with patients regarding delivery times. Some providers specifically requested that the pharmacy share tracking information with both study participants and the clinic. One respondent said:

*“… sometimes the patients would say, ‘I don’t know where my package is, it says it’s delivered, it’s not here’ and then they’d find it in some random place in there, like ‘oh it was by a plant or by the other door’… it’s kind of anxiety-inducing to have to hold the patient’s hand through that process, but it all worked out in the end…*” (ID11, abortion clinic).

Another respondent mentioned managing technical difficulties that arose while sending prescriptions: “…*for some reason, our fax was not transmitting faxes. So, we had I think two patients that were delayed, but we just didn’t realize that the fax hadn’t gone over. But they were extraordinary. You call [the pharmacy], they answered right away*” (ID18, primary care clinic). Others discussed the need to keep in mind time zone differences between the clinic and pharmacy and limited weekend business hours for the pharmacy and shipping companies when discussing a plan with participants. One provider said, “…*[the pharmacy] is an hour ahead of us, often that wasn’t enough time, based on the patient’s appointment time and everything, to actually get the meds out that da*y” (ID17, abortion clinic).

Some providers worried that mail-order dispensing may not be a realistic option for all patients due to logistical constraints, including limited financial resources or lack of stable mailing address. One provider said, “*I think my concerns with the mifepristone by mail right now, again, comes back to the payment piece. So, if insurance doesn’t cover it, or Medicaid doesn’t cover it, then it’s only available to the people who can pay out of pocket for it or who figure out that way*” (ID10, abortion clinic). Another said, “*I think for the patients that don’t have a reliable mailing address, obviously harder for patients on the go. We serve a lot of … patients who aren’t always in the same place over time, and so, I think also for them hard to get mail*” (ID2, primary care clinic).

Finally, providers were concerned that the mailing process may put patient confidentiality at risk, though most concerns about privacy appeared to be theoretical rather than based on experiences in the study. One provider said:

“*It’s always just going to be the ability for these people to get their medications in a discreet way, for people who are living with their abusers. People in situations where maybe it’s an 18-year-old but she still lives with her parents and she doesn’t want them to … It’s just always going to be the more discreet, the better. That was always my concern. Making sure that nothing else is being mailed. Like no follow-up anything with a name on it or a procedure on it or anything like that.*” (ID13, abortion clinic).

Another said, “*the biggest risk with mail-order pharmacy is that someone who’s not supposed to know about this receives the medication or opens it up…[delivering to incorrect addresses] creates a lot of anxiety and a lot of tension, especially in people who live in rural areas…*” (ID19, abortion clinic). One provider who said she was worried about privacy admitted she did not encounter any issues:

“*Thankfully I didn’t have any patients who had concerns that like, ‘oh my partner will find the package, and I’ll be in a safety issue [if] someone in my house finds the package that’s not me or if it gets intercepted somehow’, but that was certainly always in the back of my mind, like what am I going to do besides say like ‘can it be mailed to a friend or family member, or here to the clinic’ in the situation that someone has a safety concern, if their partner didn’t know they were pregnant and wouldn’t have been supportive potentially in a really dangerous way.*” (ID5, primary care clinic).

### Clinical concerns

A minority of providers expressed some clinical concerns about the mail-order pharmacy dispensing model. One primary care provider was concerned that the mail-order model may only be appropriate for early medication abortion patients and not for those close to the 70-day (or 10-week) gestational duration limit, “*I did have one patient too, who I think was like eight and a half weeks, so I was like, oh, we’re getting close …particularly because sometimes I’d be seeing these patients on a Friday, so it’s like is [the medicine] going to get to them?*” (ID5, primary care clinic). Others were concerned about the possibility that patients may not take the medication when instructed or may give it to a friend. One provider said, “*That it’s within the week gestation, that they take the medication when they say they’re going to take it, and they don’t just not take it and hold on to it…and maybe give it to a friend*” (ID12, abortion clinic). Another mentioned discomfort with the potential application of mail-order pharmacy dispensing to a fully remote model of care and consequently relying on history alone to determine eligibility, “*we share the anxiety that somebody might look at a calendar and realize that they’re 13 weeks but say they’re 9 weeks… there’s limits to how much liability to want to put as a provider on your license, you know?*” (ID20, abortion clinic).

However, most providers were pleasantly surprised that they did not encounter any clinical issues with implementing the mail-order dispensing model. An experienced abortion provider said, “*We would have concerns sometimes. Like when are people going to get their medication? When are they going to be taking their miso in a timely way after swallowing their mifepristone? …But for the most part, we had less snafus than we were anticipating*” (ID21, abortion clinic).

### Legal concerns

Most interviews were conducted just after the *Dobbs* Supreme Court decision, which removed federal protections for abortion, was leaked and then officially released in June 2022 (recruitment for many sites completed in May 2022). As a result, some providers expressed uncertainty about implementing mail-order dispensing in a rapidly changing political and legal environment around abortion in the US. One provider mentioned that mail-order pharmacy dispensing would need to be further adapted to consider state laws as well as the REMS, “*Because of the … regulations in [our state], there’s certain things that we need to do even outside of the REMS*” (ID10, abortion clinic). Another said their priority was to manage the challenges they were currently facing trying to take care of patients from out of state and implied that prescribing abortion pills across state lines was legally risky:

“*Right now we have volunteers in the building just to be picking up the phones, because it is so, so nonstop. We’re fielding hundreds of calls every day. Our call log to get back to people to make appointments is like 350 people deep right now. I really can’t overestimate how intense things are getting immediately…. It’s pretty much all [out of state] people at this moment who are coming in. And we can’t prescribe across that state line. Like we wouldn’t be able to send medication there.*” (ID13, abortion clinic).

Another provider also expressed concern about increasing legal regulation of prescribing and shipping abortion pills across state lines:

“*I am worried that we’re going to start to see restrictions around [the mail-order pharmacy], is [the pharmacy home state] going to state a law that says they can’t ship it outside of the state, and now I’m in …a tiny fricking state that does not exactly have like a big mail-order pharmacy within the state lines, or am I going to have to be concerned that a patient has to be able to go to [the city], or go to [a neighboring state], or have a mailing address there if we start to see more regulations around this?*” (ID5, primary care clinic).

One respondent revealed legal concerns about cross-state provision of care; she said, “*with so many people traveling to us from hostile states … we are going to just have them take it right then and there. So, there is no question about where they’re taking the mifepristone*” (ID17, abortion clinic).

### Challenges integrating abortion into primary care

Respondents from primary care clinics described some of the challenges they faced attempting to integrate abortion into their scope of practice prior to the study and what they anticipated facing once the study ended. These included lack of support from administrative leadership, limited freedom in determining specific clinical protocols, minimal training in abortion care, and confusion around payment and billing for medication abortion services. One provider reflected on her experience seeking approval from administrative leadership, “…*it really comes down to whether someone just says no, and just sort of stops you in your tracks, like somebody higher up than you who you have to listen to. Then the other thing is whether there’s obstructions that just take so much time that you can’t make it a priority because you’re a busy primary care doctor*” (ID1, primary care clinic). Another primary care provider obtained administrative support without issue, but said she had little freedom to implement clinical protocols consistent with the latest evidence: “*I would’ve loved to have been able to offer having betas drawn or doing a follow up pregnancy test, instead of necessarily having to bring patients back for ultrasounds, but because that’s what our [obstetrics] group does, they basically said you have to have this done…*” (ID5, primary care clinic).

One respondent highlighted a lack of training in abortion as a potential challenge in integrating the mail-order model into primary care settings, saying, “*the biggest limitation was probably training. We didn’t get that as part of our training as a resident in pediatrics …*” (ID3, primary care clinic). In addition, several respondents new to abortion care highlighted their confusion around payment and billing. One said, “*the main issue is going to be funding now… of course when you’re thinking about practically integrating medication abortion, that’s such a huge barrier is the billing piece of it. … most of the patients in our clinic have Medicaid or state insurance. And so administration felt that we would need to have a financial counselor on site at our clinic in order to offer this service*” (ID15, primary care clinic). To make integrating abortion into primary care settings smoother, some respondents recommended developing electronic health record templates or smart phrases for new providers to use when prescribing and monitoring medication abortion patients.

## Discussion

Findings from this qualitative study highlight that mail-order pharmacy dispensing of mifepristone is acceptable to both primary care and abortion providers, supporting recent demonstrations of the effectiveness, safety, and acceptability to patients of this care model [15]. We found that staff and clinicians at participating primary care and abortion clinics were hopeful that mail-order pharmacy dispensing could improve abortion access and enable more providers to offer the service. Participants at both primary care and abortion sites agreed that offering mail-order pharmacy dispensing would contribute to normalizing abortion care. These findings confirm the supportive perspectives of a sample of Illinois providers in a study that asked about hypothetical pharmacy dispensing of mifepristone [18].

Providers in our study agreed that mail-order pharmacy dispensing should be one of many options available to patients to improve access to medication abortion, including fully remote medication abortion (with telehealth evaluation for eligibility and the mailing of medications), use of a brick-and-mortar pharmacy, and use of a mail-order pharmacy after in-person evaluation for eligibility. Despite recent announcements that two of the largest US brick-and-mortar pharmacy chains will begin dispensing mifepristone in a handful of states in March 2024, uptake of the brick-and-mortar model has been slow, and many brick-and-mortar pharmacies do not yet stock mifepristone. Only 21 pharmacies have elected to notify the public of their commitment to dispensing mifepristone [19]. Even where brick-and-mortar dispensing is available, the mail-order model may be preferable for patients who have concerns about confidentiality and privacy [13]. In a recent qualitative study about interest in alternative models of medication abortion provision, some participants worried they might be recognized by someone they know when picking up abortion medications at a pharmacy [20]. Some people, including young people and people without abortion experience, may prefer to see a provider and receive their medications in person [21]. Clinicians will have to evaluate the size and needs of their particular patient population and resource constraints to decide which models to offer. Clinicians in this study with relatively high volumes of abortion patients may benefit from continuing to stock mifepristone on-site for patients who prefer to obtain it in a clinic setting, in addition to offering the mail-order pharmacy dispensing option. The model we studied is likely most relevant for primary care providers or other clinicians, including private ob/gyns, who provide care for a small volume of medication abortion patients and who likely face challenges initiating the stocking and tracking of mifepristone on-site.

Our findings indicate the potential for mail-order pharmacy dispensing of mifepristone to support the integration of abortion care into primary care and other outpatient settings, ultimately improving access and quality of care. A national survey of obstetrician-gynecologists conducted in 2016–2017 found that the removal of the in-person dispensing requirement could potentially double the number of medication abortion providers in the US [22]. Primary care providers, including advanced practice clinicians, are well equipped to provide patient-centered counseling, early pregnancy evaluation, and miscarriage management. Primary care providers can routinely provide safe and effective medication and aspiration abortion care [23, 24]. Furthermore, patients appreciate obtaining abortion care in primary care settings, where there is privacy, convenience, and continuity of care [25].

Providers in this study highlighted that the largest improvements in abortion access due to mail-order pharmacy dispensing would be seen when the model is integrated with telemedicine to enable fully remote care for patients who cannot or prefer not to visit a clinic in person. Fully remote care models – that is, telehealth evaluation for medication abortion eligibility followed by mailing abortion pills—have recently become recognized by the US FDA as a standard approach to care [2]. Telehealth abortion care has been shown to be highly acceptable and effective, with benefits to patients including privacy and expediency [26–28]. A study conducted during the COVID-19 pandemic in New Jersey, New York, and Washington found that a single family physician could successfully provide medication abortion services to the entire state using asynchronous online consultations and medications mailed directly to patients through the online platform Aid Access [29].

Despite recent regulatory changes enabling the provision of medication abortion by telehealth and mail-order pharmacy dispensing, the US abortion policy environment is rapidly changing, and there is uncertainty as to whether and where these new models of care can be implemented. Even in states where abortion remains legal after the Supreme Court’s decision in *Dobbs*, additional state laws require some or all of the medication abortion process to take place in person. Currently, 24 states plus DC allow telehealth to be used to provide medication abortion [30]. Institutional and organizational policies in some settings prevent clinicians from providing medication abortion via telehealth even in states where the law permits it, due to anxiety around liability, lack of infrastructure or funding, or an unwillingness to adopt new care models [8, 31]. As a growing number of states adopt protections for abortion providers who offer telehealth care to residents of other states, in what are referred to as “shield laws” [32], such barriers hopefully will diminish.

Providers in our study acknowledged that mail-order pharmacy dispensing may not be feasible for all patients, even where laws permit it. Socioeconomic barriers to healthcare access, lack of stable housing, and health literacy inequities may prevent patients from utilizing a mail-order option. And given that health insurance, especially Medicaid, typically does not pay for express shipping, there may be financial barriers patients must overcome to use the mail-order option effectively. Moreover, to ensure smooth implementation, insurances will also need to add mifepristone as a pharmacy benefit so the pharmacy can bill directly for the drug and dispensing costs.

A limitation of this study is that it included only providers at clinics that opted to participate in a study of mail-order pharmacy dispensing of mifepristone. Results do not reflect the opinions and experiences of providers at sites that were not able to obtain administrative support to participate in the study or were not motivated to try to integrate abortion care into their services. We were also unable to effectively assess differences in themes across provider roles (administrators versus clinicians), given that roles were not standardized across clinics; some clinicians were involved in consenting participants, and some were not, and some administrators had clinical training and others did not. However, this study is strengthened by the inclusion of perspectives from both primary care and abortion providers across seven states.

## Conclusions

Mail-order pharmacy dispensing enabled primary care and abortion providers to better meet the needs of more patients and facilitated the introduction of abortion care in primary care settings. Participating staff and clinicians were hopeful about the potential of the mail-order model to improve access to abortion services and to enable more providers to offer abortion care. Providers recognized that mail-order pharmacy dispensing may not be the right option for all individuals and should be one of many options for accessing care, particularly in the context of legal uncertainty surrounding medication abortion provision after the Supreme Court’s *Dobbs* decision constrained care in many states.

### Electronic supplementary material

Below is the link to the electronic supplementary material.

Supplementary Material 1

Supplementary Material 2

## Data Availability

The datasets generated and/or analyzed during the current study are not publicly available due to privacy concerns but relevant portions may be available from the corresponding author on reasonable request.
