# Supplementary material for: Primary care and abortion provider perspectives on mail-order medication abortion: a qualitative study
Source: BMC Womens Health. 2024 Jul 3;24:382. doi: 10.1186/s12905-024-03202-z (PMC11221167; doi:10.1186/s12905-024-03202-z)
Supplement: Supplementary file 1 — Supplementary Material 1 [file 12905_2024_3202_MOESM1_ESM.docx]

**Supplement 1**

**Provider Interviews:**

**Alternative Provision of Medication Abortion via Mail-order Pharmacy Dispensing**

**For sites that began providing abortion care as part of the study:**

1. Tell me about the clinic and your role on the team. (clinic type, focus, general patient population)
   1. Can you tell me a little about your prior experience, if any, with abortion care?
   2. To what extent do you have experience mailing medications or working with mail-order pharmacies for any medication?
   3. Can you tell me a little about why your clinic had not been providing medication abortions prior to the study?
2. Why did you or your clinic elect to participate in this study?
3. Tell me about the process of getting your clinic ready to offer medication abortion services by participating in this mail-order study.
4. Walk me through what a typical enrollment/prescribing experience was like during this study.

[**Focus on how patients reacted, how easy/hard the study activities were, whether patients had a lot of questions or concerns related to medication abortion**]

- 1. What was your role in the study process (recruitment/enrollment/prescribing)
     1. How often did you participate in the recruitment and/or prescribing process?
  2. Can you tell me about any challenges you faced specifically with study activities?

1. Can you tell me about how you felt providing medication abortion during this study?
2. Can you describe your experience with any barriers or challenges integrating medication abortion into your practice?
   1. How did you deal with/overcome these barriers?
   2. Tell me about your experience with any barriers or challenges with mail-order dispensing and how you/your clinic dealt with them.
3. Please describe any challenges offering mail-order dispensing of mifepristone that you felt you or your practice was unable to overcome, if any. Please focus on challenges related to providing the service (as opposed to participating in the research study).
4. Can you tell me about anything that surprised you about offering medication abortion to your patients and/or the mail order model?
   1. In your experience, how did patients feel about mail order model?
5. Given your experience so far, what kind of training and support do you think would be most helpful for providers/staff to begin providing medication abortion?

- 1. What support, if any, did you use when providing medication abortion in the study? By support, we mean anything from reaching out to the study team or other colleagues with questions, using resources from other organizations like NAF or RHAP, referring to journal articles, etc.
  2. What additional (internal or external) training or support would have been helpful to have? And why?
  3. **Additional probe if needed**: What training/support would be helpful specifically for counseling patients, determining eligibility for medication abortion, prescribing to mail-order pharmacies, or following up with patients?

1. Can you tell me more about the experience of working with a mail-order pharmacy to dispense MAB medications for this study?
   1. What worked well?
   2. What could be improved?
2. What were the benefits (to patients/providers/others), if any, of offering mail-order dispensing of mifepristone compared to having mifepristone on site and dispensing directly to patients?
3. What concerns do you have, if any, about the mail-order model for medication abortion outside the context of the study?
   1. What could be improved about the mail-order model?
   2. Concerns for patients?
   3. Concerns for providers?
   4. Concerns for administrators?
   5. Concerns for billers?
4. Do you think you will continue to offer medication abortion using a mail-order pharmacy?
   1. If no, why not?
   2. If yes, how do you plan to provide the service? (probe about the use of telemedicine vs. evaluating patients in person before sending the prescription)
5. Is there anything else you’d like to share about providing medication abortion or about mail-order pharmacy dispensing of mifepristone?
