# Supplementary material for: Primary care and abortion provider perspectives on mail-order medication abortion: a qualitative study
Source: BMC Womens Health. 2024 Jul 3;24:382. doi: 10.1186/s12905-024-03202-z (PMC11221167; doi:10.1186/s12905-024-03202-z)
Supplement: Supplementary file 2 — Supplementary Material 2 [file 12905_2024_3202_MOESM2_ESM.docx]

**Supplement 2**

**Provider Interviews:**

**Alternative Provision of Medication Abortion via Mail-order Pharmacy Dispensing**

**Sites already providing abortion:**

1. Tell me briefly about your training and your background in abortion care.
   1. Tell me about any prior experience you have with mailing medications (including medication abortion) or working with mail-order pharmacists.
2. Please describe your role in the provision of abortion care at your facility.
3. Please describe your role in the research study.
   1. How many times were you involved with the recruitment or care of a study patient throughout the study? Tell me about that.
4. Walk me through what a typical enrollment and/or prescribing experience was like for you during this study?

[**Focus on how patients reacted, how easy/hard the study activities were, whether patients had a lot of questions or concerns related to medication abortion**]

- 1. What, if anything, surprised you about offering mifepristone by mail?

1. What kind of training and/or support do you think would be most helpful for providers/staff like you who are interested in offering the mail-order model?
   1. What support or resources, if any, did you use to ensure the mail-order model was successful during the study? (examples may include reaching out to the study team or colleagues in the field with questions, resources from other organizations like NAF or RHAP, journal articles, etc.)
   2. What additional training or support would be helpful to have when implementing the mail-order model?
2. Can you tell me more about the experience of working with a mail-order pharmacy to dispense mifepristone and misoprostol for this study?
   1. What worked well?
   2. What could be improved?
3. What were the benefits (to patients/providers/others), if any, of offering mail-order dispensing of mifepristone compared to having mifepristone on site and dispensing directly to patients?
4. What concerns do you have, if any, about the mail-order model outside the context of the study?
   1. What could be improved about the mail-order model?
   2. Concerns for patients?
   3. Concerns for providers?
   4. Concerns for administrators?
   5. Concerns for billers?
   6. Can you tell me about any challenges you faced specifically with study activities?
5. Do you think you will continue to offer medication abortion using a mail-order pharmacy?
   1. If no, why not?
   2. If yes, how do you plan to provide the service? (probe about the use of telemedicine vs. evaluating patients in person before sending the prescription)
6. Is there anything else you’d like to share about providing medication abortion or about mail-order pharmacy dispensing of mifepristone?
